# Novel characterization discoveries of ferroptosis-associated molecules in COAD microenvironment based TCGA data

**DOI:** 10.3389/fmolb.2022.1102735

**Published:** 2022-12-13

**Authors:** Salem Baldi, Yun He, Igor Ivanov, Yaping Sun, Wei Feng, Moath Refat, Shadi A. D. Mohammed, Salah Adlat, Zixuan Tian, Yi Wang, Yaping Gao, Hui Tian

**Affiliations:** ^1^ Research Center of Molecular Diagnostics and Sequencing, Axbio Biotechnology (Shenzhen) Co., Ltd., Shenzhen, China; ^2^ Research Center of Molecular Diagnostics and Sequencing, Research Institute of Tsinghua University in Shenzhen, Shenzhen, China; ^3^ Department of Biochemistry and Molecular Biology, The Key Laboratory of Environment and Genes Related to Disease of Ministry of Education, Health Science Center, Xi’an Jiaotong University, Xi’an, China; ^4^ Graduate School of Heilongjiang University of Chinese Medicine, Harbin, China; ^5^ Department of Gastroenterology and Hepatology, Mayo Clinic, Rochester, MN, United States

**Keywords:** COAD microenvironment, metastasis, ferroptosis, immune cell infiltration, TCGA

## Abstract

**Background and Objective:** One of the most recent forms of programmed cell death, ferroptosis, is crucial in tumorigenesis. Ferroptosis is characterized by iron-dependent oxidative destruction of cellular membranes following the antioxidant system’s failure. However, it is unknown whether ferroptosis-related genes (FRGs) are associated with colon adenocarcinoma (COAD) metastasis, immune cell infiltration, and oxidative stress in COAD. The current study concentrated on FRGs expression in colon cancer metastasis, their relationship to immune cell infiltration (ICI), and potential pathological pathways in COAD.

**Methods and Results:** Clinical information and mRNA expression patterns for patients with COAD metastasis were obtained from the public TCGA database. Patients with low mRNA levels showed good overall survival than patients with high mRNA levels. The genomic-clinicopathologic nomogram was subsequently created by combining risk score and clinicopathological features. Absolute Shrinkage and Selection Operator have shown a 4 gene signature that can stratify cancer patients into high-risk versus low-risk. These four FRGs were found to be significantly linked to the overall survival of COAD patients and predicted high risk score. Next, age, stage, and PTNM were combined in univariate and multivariate cox regression models to perform a filtering procedure. The receiver operating characteristic (ROC) and calibration curves indicated that constructed signature model exhibited high prediction accuracy and clinical relevance in COAD. ARID3A showed a strong negative correlation with a wide range of immune tumour-infiltrating cells in COAD microenvironment. According to the single sample gene set enrichment analysis (ssGSEA) results, FRGs are involved in variety of pathological pathways including PI3K-AKT-mTOR pathway, reactive oxygen species (ROS) pathway, response to hypoxia pathway, and other inflammation related pathways. Moreover, dysregulation of FRGs in COAD patients showed a significance correlation with wide range of miRNAs and transcription factors (TFs).

**Conclusion:** We identified new diagnostic biomarkers and established prognostic models for ferroptosis related programmed cell death in COAD metastasis. FRGs may improve tumor cell survival by activating the TGFB pathway, which can stimulate ROS production, accelerates ECM breakdown, and promote tumor progression and invasion. Genes implicated in ferroptosis, as revealed by the Kaplan Meier and a genomic-clinicopathologic nomogram, are potential therapeutic targets and prognosis indications for metastasis COAD patients.

## 1 Introduction

### 1.1 Colon cancer

Colon cancer is the third most frequently diagnosed cancer in males and females worldwide, with 80, 690 (8%), 70,340 (8%) new cases and 28,400 (9%), 24,180 (8%) deaths in male and female, respectively (Siegel et al., 2022). The high incidence rate or mortality is because of the lack of early detection and the fact that it is often diagnosed in its later stages (Zhou et al., 2019). The 5-year overall survival (OS) rate for COAD patients is still low, despite the availability of various targeted medicines and immunotherapies in recent years. Therefore, studying the molecular mechanism of the occurrence and development of colorectal cancer is an important subject of clinical research. Preventive measures, such as screening and finding new therapeutic targets, are also critical to improve patients’ survival and prognosis of colon cancer.

### 1.2 Iron death

Various human diseases can be prevented by targeting regulated cell death, which includes necroptosis, pyroptosis, ferroptosis, entotic cell death, lysosome-dependent cell death, and autophagy-dependent cell death. Ferroptosis is iron-dependent programmed cell death, first postulated by Dixon in 2012; it differs from apoptosis, pyrolysis, and autophagy at levels of cell morphology, biochemical features, and regulation and occurs through Fe(II)-dependent lipid peroxidation to insufficient cellular reducing capacity (Dixon et al., 2012; Jiang et al., 2020). Several studies have connected ferroptosis to cancer development and progression (Xia et al., 2019; Jiang et al., 2020). Since tumor cells can maintain or acquire ferroptosis sensitivity while surviving cell death, ferroptosis therapy for cancer is gaining attention. Wei et al. found that small molecule drugs that activated p53 had potent inhibitory action against HCT116 cells by inducing ferroptosis (Wei et al., 2018). Combinatorial therapy with ferroptosis medicine and tumor necrosis factor-related apoptosis-inducing ligands led to synergistic apoptosis and growth regression of CRC (Lee et al., 2019). Thereby, ferroptosis-related genes (FRGs) are very significant in cancer patients (Jiang et al., 2015; Ou et al., 2016; Bersuker et al., 2019; Doll et al., 2019; Li et al., 2020) and could be promising therapeutic targets and prognostic indicators in colon adenocarcinoma (COAD).

### 1.3 Working hypothesis

Our working hypothesis was that FRGs promotes colon cancer metastasis and play a role in oxidative stress.

### 1.4 Study design

Based on above mentioned hypothesis we developed prognostic model, validated, and explored the mechanism by which FRGs promotes COAD progression and invasion. Our results showed that there are solid predictive genes and offered a novel, personalized approach to treating COAD.

## 2 Materials and methods

### 2.1 Data acquisition and identification of differentially expressed genes (DEGs)

Ferroptosis-related genes were derived from Ze-Xian Liu et al. RNA-sequencing expression (level 3) profiles and corresponding clinical information for COAD were downloaded from the TCGA dataset (https://portal.gdc.com). R package, version 4.0.3 was used to implement the analysis. Genes with a *p*-value of less than 0.05 and log FC > 1 were chosen for further investigation. Only patients who had M1 metastases and higher were considered for participation in the trial according to the inclusion criteria for patient selection.

### 2.2 Nomogram construction

The Cox regression analysis was conducted to determine if risk scores and relevant clinical indicators could be identified as prospective predictors of OS for COAD patients. Based on the Cox regression analysis results, a prognostic nomogram was built using the stepwise Cox regression model to predict the 1, 3, and 5-year OS of COAD patients included in the TCGA dataset. This was done to determine the probability of survival. The area under the curve was used to evaluate the nomogram’s ability to discriminate between categories (AUC). Using the calibration curve, a graph comparing the expected OS of the nomogram to the observed survival rates was constructed.

### 2.3 Correlation between genes and pathways in COAD

The genes found in the associated pathways were gathered and examined using the R software’s GSVA package, with the parameter method set to the ssGSEA algorithm’ as the final step. Finally, Spearman correlation was used to examine the correlation between the genes and the pathway score. The analysis methods and R packages were implemented by R version 4.0.3. *p*-value < 0.05 was considered statistically significant.

### 2.4 Regulatory network of FRGs

We predicted the potential ironoptosis-associated non-coding RNAs by performing co-expression analysis with identified iron-related genes in COAD. We predicted miRNAs that target FGRs using the GSCA-hosted TCGA database (GSCA - Gene Set Cancer Analysis (hust.edu.cn). We then utilized the knockTF database to identify FRGs-specific transcription factors (TFs) that significantly influence FGRs based on expression and ChIP-seq/motif evidence (KnockTF-Search (licpathway.net)).

## 3 Results

### 3.1 Identification of DEGs related to ferroptosis in the TCGA cohort

**FIGURE 1:**
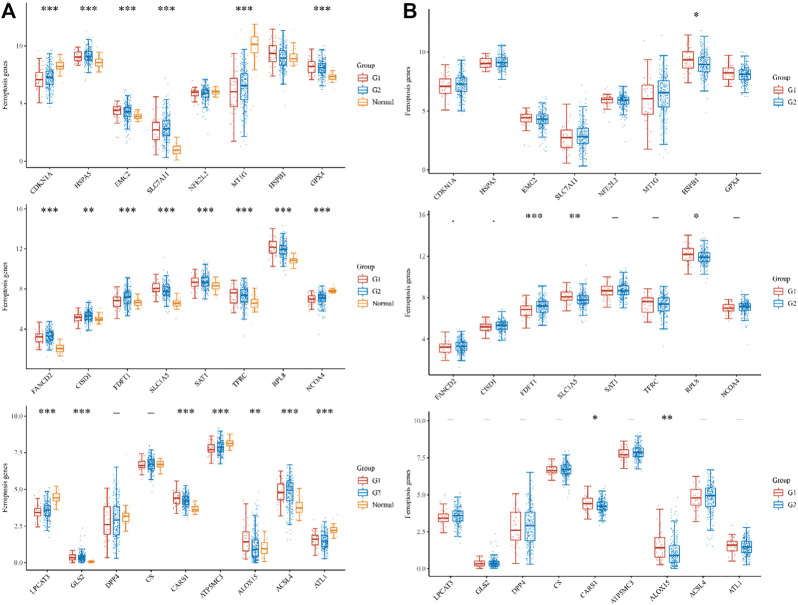
The expression distribution of ferroptosis-related mRNA in tumor tissues and normal tissues. **(A)** histogram shows the dysregulation of iron death in metastasis G1, non-metastases G2, and Normal samples, respectively. **(B)**histogram shows the number of significantly differentially expressed genes in metastasis G1, non-metastases G2, respectively. *p < 0.05, **p < 0.01, ***p < 0.001.

In total, 426 sample, 52 metastases, 333 non-metastases COAD patients and 41 normal samples from TCGA cohorts were considered for inclusion in the study (Supplementary Table S1). We investigated RNA-seq data from the TCGA dataset to determine the expression differences of ferroptosis-related genes between tumor tissues and neighboring normal tissues. Among the 25 ferroptosis-related genes that were investigated, tumor tissues and adjacent normal tissues displayed significant differences in 21 genes. CDKN1A, HSPA5, EMC2, SLC7A11, MT1G, GPX4, FANCD2, CISD1, FDFT1, SLC1A5, SAT1, TFRC, RPL8, NCOA4, LPCAT3, GLS2, CARS1, ATP5MC3, ALOX15, ACSL4, and ATL1 (Figure 1A). To be more specific the differential expressed gene in metastases and non-metastases tissues were identified. Among the above-mentioned genes, the following genes were identified FDFT1, SLC1A5, RPL8, CARS1, and ALOX15 in addition to HSPB1.

### 3.2 Prognostic genes identified in the TCGA COAD cohort

**FIGURE 2:**
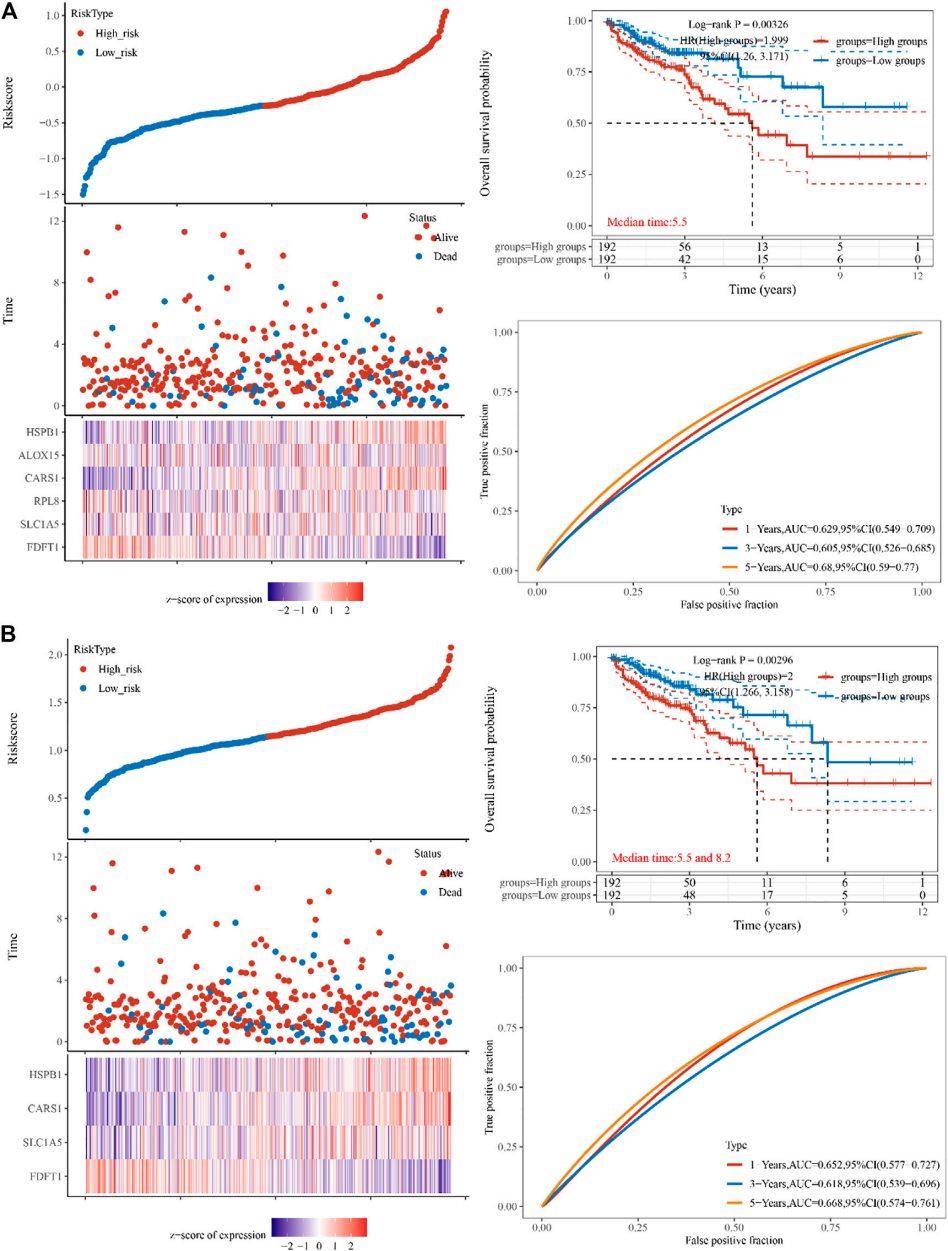
Constructing the TCGA cohort’s genes. **(A)** The multi-factor cox regression analysis showed six prognosis related genes in colon cancer **(B)** The forest analysis showed four prognosis related genes in colon cancer using 10x cross-validation (*p* < 0.05) The top scatters represent TCGA cohort’s risk score distribution from low to high. Kaplan-Meier survival analysis of the risk model from dataset, comparison among different groups was made by log-rank test. HR (High exp) represents the hazard ratio of the low-expression sample relatives to the high-expression sample. HR > 1 indicates the genes are a risk factor, and HR < 1 indicates the genes are a protective factor. HR (95%Cl). Heatmap is the gene expression from the signature. Prognostic performance was evaluated using the AUC of the time-dependent ROC curve analysis for OS in the TCGA cohort.

To reduce dimensionality and construct prognostic models based on Cox and lasso regression methods, iron death-related gene difference analysis results were utilized to evaluate the prognosis of these genes on CAOD tumor patients. Based on the median cut-off value, high- and low-risk patients were separated into two groups. The following formula was used to determine the risk score of the outcome = $\overset{n}{\underset{i=1}{\sum }}\left(coefi\times Expri\right)$ . The prognostic model constructed using multi-factor cox regression analysis is represented in Figure 2A. AIC = 793.6083 Riskscore= (-0.3887)*FDFT1+(-0.2156)*SLC1A5+(-0.0725)*RPL8+(0.6781)*CARS1+(-0.0524)*ALOX15+(0.253)*HSPB1. LASSO regression identified (FDFT1, HSPB1, SLC1A5, and CARS1 a signature model gene. lambda. min = 0.0151, (Riskscore= (-0.2482) *FDFT1+(-0.03) *SLC1A5+(0.4137) *CARS1+(0.1543) *HSPB1 (Figure 2B).

### 3.3 Independent prognostic value of the FRGs gene in COAD

**FIGURE 3:**
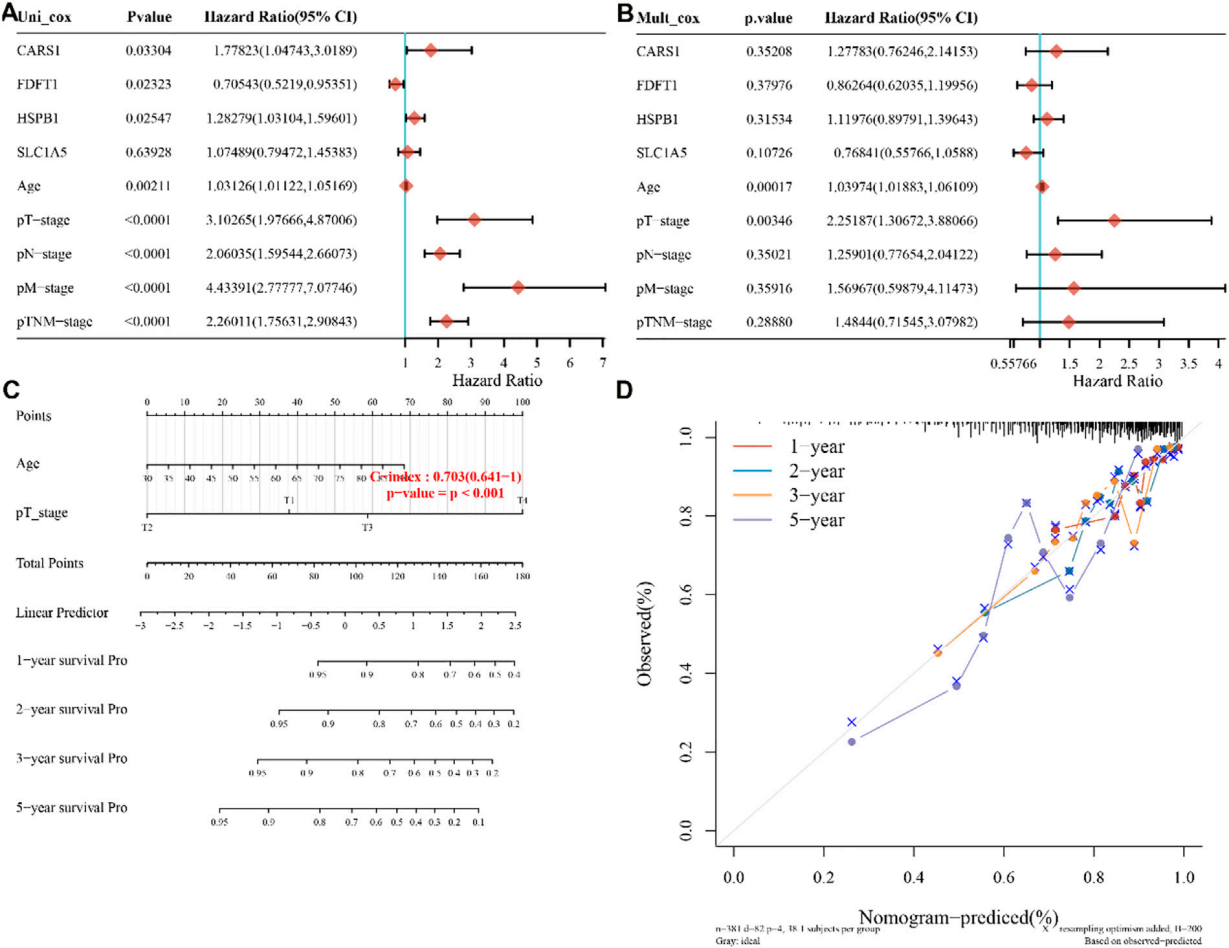
four-genes prognostic value and predictive nomogram. **(A,B)** Multivariate and univariate Cox regression analysis of the four genes is both available, **(C)** Nomogram development based on independent prognostic value for the 4-genes, **(D)** Predicting 1–5-year survival using a nomogram. pT,pN, pM are pathological stages where T is the stage (generally related to the size and depth of the tumor), N is lymph node metastasis, and M is distant metastasis. pTNM stands for Pathological tumor-node-metastasis (pTNM) staging.

The TCGA dataset was used to do univariate and multivariate Cox regression analysis on the identified 4 genes signature to determine whether the ability of the prognostic significant in predicting OS was independent. The effect of the 4 genes and clinical factors on prognosis was investigated with the help of the Cox regression analysis. These factors included age, pT, pM, pN, and Pathological tumor-node-metastasis (pTNM) staging. The variable was significant in the univariate Cox regression analysis and was thought to be related to prognosis (Figure 3A). Multivariate Cox regression analysis found these variables to be non-significant, except for age and pT stage and of the four discovered genes, CARS1 and HSPB1 had a hazard ratio greater than 1 (Figure 3B). Based on the independent prognostic factors for the OS of multi-factor Cox regression analysis, we developed a nomogram for predicting 1, 2, 3, and 5-year survival rates (Figures 3C, D). In the TCGA dataset, the calibration curve for the probability of 1, 2, 3, and 5-year OS exhibited the best agreement between observation and prediction. After bias adjustment, the C-index was found to be 0.703, *p* < 0.001 indicating a strong level of clinical diagnosis performance for the model signature genes. As a result, the identified ferroptosis-related gene impacts the prognosis of COAD and may serve as a possible diagnostic factor for COAD patients.

### 3.4 Characterization of ferroptosis-associated molecules in COAD microenvironment

The enrichment fraction of each sample on each pathway was calculated in turn using the ssGSEA algorithm to obtain the connection between the sample and the pathway, and we then obtained the relationship between the gene and the pathway by calculating the correlation between gene expression and pathway score using spearman analysis. The tumor cell proliferation signature score was found to be positively correlated with CARS1 and FDFT1, indicating that the signature model has a high proliferation rate. Furthermore, we found a statistically significant activation of the cellular response to hypoxia signature and the G2M checkpoint in SLC1A5, CARS1, and FDFT1 (Table1).

**TABLE 1:** Pathways correlated to FGRs genes in COAD.

Symbol	Pathway	Cor	*p*_value
FDFT1	Tumor_Inflammation_Signature	−0.05	0.321
FDFT1	Cellular_response_to_hypoxia	0.23	7.39e−07
FDFT1	Tumor_proliferation_signature	0.31	1.22e−11
FDFT1	EMT_markers	−0.06	0.177
FDFT1	ECM−relatted_genes	−0.08	0.091
FDFT1	Apoptosis	0.08	0.102
FDFT1	Angiogenesis	−0.04	0.376
FDFT1	DNA_repair	0.12	0.011
FDFT1	G2M_checkpoint	0.020275	8.36e−08
FDFT1	Inflammatory_response	0.06	0.174
FDFT1	PI3K_AKT_mTOR_pathway	0.05	0.283
FDFT1	P53_pathway	0.19	3.26e−05
FDFT1	MYC_targets	0.20	1.24e−05
FDFT1	TGFB	−0.08	0.106
FDFT1	IL−10_Anti−inflammatory_Signaling_Pathway	0.11	0.022
FDFT1	Genes_up−regulated_by_reactive_oxigen_species_(ROS)	0.09	0.063
FDFT1	DNA_replication	0.19	3.36e−05
FDFT1	Collagen_formation	−0.09	0.063
FDFT1	Degradation_of_ECM	−0.03	0.533
FDFT1	Ferroptosi	0.07	0.123
CARS1	Tumor_Inflammation_Signature	−0.05	0.321
CARS1	Cellular_response_to_hypoxia	0.18	1.48e−04
CARS1	Tumor_proliferation_signature	0.24	1.74e−07
CARS1	EMT_marker	0.09	0.066
CARS1	ECM−relatted_genes	0.05	0.254
CARS1	Angiogenesis	0.09	0.046
CARS1	Apoptosis	0.08	0.089
CARS1	DNA_repair	0.26	1.59e−08
CARS1	G2M_checkpoint	0.34	5.04e−14
CARS1	Inflammatory_response	0	0.958
CARS1	PI3K_AKT_mTOR_pathway	0.23	7.03e−07
CARS1	P53_pathway	0.03	0.57
CARS1	MYC_targets	0.33	2.85e−13
CARS1	TGFB	0.04	0.381
CARS1	IL−10_Anti−inflammatory_Signaling_Pathway	−0.01	0.896
CARS1	Genes_up−regulated_by_reactive_oxigen_species_(ROS)	−0.04	0.432
CARS1	DNA_replication	0.3	3.21e−11
CARS1	Collagen_formation	0.09	0.06
CARS1	Degradation_of_ECM	0.08	0.082
CARS1	Ferroptosi	0.19	6.32e−05
HSPB1	Tumor_Inflammation_Signature	−0.06	0.237
HSPB1	Cellular_response_to_hypoxia	0.02	0.716
HSPB1	umor_proliferation_signature	−0.25	5.2e−08
HSPB1	EMT_markers	0.28	1.4e−09
HSPB1	ECM−relatted_genes	0.13	0.005
HSPB1	Angiogenesis	0.19	4.91e−05
HSPB1	Apoptosis	0.09	0.063
HSPB1	DNA_repair	−0.03	0.491
HSPB1	G2M_checkpoint	−0.26	1.87e−08
HSPB1	Inflammatory_response	0.03	0.526
HSPB1	PI3K_AKT_mTOR_pathway	0	0.991
HSPB1	P53_pathway	0.28	1.08e−09
HSPB1	MYC_targets	−0.24	2.89e−07
HSPB1	TGFB	0.14	0.003
HSPB1	L−10_Anti−inflammatory_Signaling_Pathway	0.05	0.327
HSPB1	Genes_up−regulated_by_reactive_oxigen_species_(ROS)	0.25	4.3e−08
HSPB1	DNA_replication	−0.16	0.001
HSPB1	Collagen_formation	0.24	3.05e−07
HSPB1	Degradation_of_ECM	0.21	4.03e−06
HSPB1	Ferroptosis	0.17	1.95e−04
SLC1A5	Tumor_Inflammation_Signature	−0.21	9.87e−06
SLC1A5	Cellular_response_to_hypoxia	−0.03	0.542
SLC1A5	Tumor_proliferation_signature	0.01	0.767
SLC1A5	EMT_markers	0.533	0.533
SLC1A5	ECM−relatted_genes	−0.08	0.11
SLC1A5	Angiogenesis	−0.03	0.542
SLC1A5	Apoptosis	−0.18	1.52e−04
SLC1A5	DNA_repair	0.18	1.39e−04
SLC1A5	G2M_checkpoint	0.1	0.043
SLC1A5	Inflammatory_response	−0.18	9.07e−05
SLC1A5	PI3K_AKT_mTOR_pathway	0.03	0.559
SLC1A5	P53_pathway	−0.01	0.906
SLC1A5	MYC_targets	0.11	0.021
SLC1A5	TGFB	−0.04	0.372
SLC1A5	IL−10_Anti−inflammatory_Signaling_Pathway	−0.20	1.6e−05
SLC1A5	Genes_up−regulated_by_reactive_oxigen_species_(ROS)	0.05	0.259
SLC1A5	DNA_replication	0.2	1.57e−05
SLC1A5	Collagen_formation	0.04	0.427
SLC1A5	Degradation_of_ECM	−0.03, 0.16	0.163
SLC1A5	Ferroptosis	0.657	−0.02

Note: cor, correlation.

### 3.5 FRGs correlate with immune cell infiltration in COAD

An increasing number of studies have suggested an interaction between immune response and pathophysiological processes. GSCA was performed to evaluate the correlation between FRGs and the available immune cells in COAD. FRFs correlated negatively with the infiltration of immune cells, including B cells, CD4-T, CD8-T, central memory, NKT, Tfh, Th17, Th2, and other immune cells presented in Table 2. These results indicated that dysregulation of FRGs expression is associated with worse immune cell infiltration in the COAD microenvironment.

**TABLE 2:** FRGs and immune cell infiltration.

Cancer type	Gene symbol	Cell_type	Corrrelation	*p*_value	Fdr
COAD	CARS1	Bcell	−0.21337	0.000096037	0.000327
COAD	CARS1	CD4_T	−0.19581	0.000353103	0.000764
COAD	CARS1	CD4_naive	0.029763	0.59063182	0.904695
COAD	CARS1	CD8_T	−0.12105	0.028142976	0.06217
COAD	CARS1	CD8_naive	0.045845	0.407207002	0.511177
COAD	CARS1	Central_memory	−0.15803	0.004057678	0.019381
COAD	CARS1	Cytotoxic	0.002643	0.961910357	0.973297
COAD	CARS1	DC	0.117239	0.033522256	0.074959
COAD	CARS1	Effector_memory	0.028785	0.602898028	0.727797
COAD	CARS1	Exhausted	0.189201	0.000560693	0.00198
COAD	CARS1	Gamma_delta	0.17752	0.001223319	0.004746
COAD	CARS1	InfiltrationScore	−0.06767	0.22090454	0.287603
COAD	CARS1	MAIT	−0.20101	0.000242966	0.000602
COAD	CARS1	Macrophage	0.01099	0.842588283	0.891323
COAD	CARS1	Monocyte	0.209679	0.0001274	0.00041
COAD	CARS1	NK	−0.0985	0.074396025	0.105847
COAD	CARS1	NKT	−0.12172	0.027277463	0.05036
COAD	CARS1	Neutrophil	0.092057	0.09552524	0.154205
COAD	CARS1	Tfh	−0.19943	0.000272399	0.000714
COAD	CARS1	Th1	0.172121	0.001727179	0.007843
COAD	CARS1	Th17	−0.21842	0.000064685	0.000325
COAD	CARS1	Th2	−0.19984	0.000264373	0.000571
COAD	CARS1	Tr1	−0.12894	0.019301647	0.042031
COAD	CARS1	iTreg	0.019994	0.717866105	0.79491
COAD	CARS1	nTreg	0.195468	0.000361876	0.000824
COAD	FDFT1	Bcell	−0.11041	0.045370724	0.077487
COAD	FDFT1	CD4_T	−0.09759	0.07711229	0.110747
COAD	FDFT1	CD4_naive	0.005977	0.913988751	0.975471
COAD	FDFT1	CD8_T	0.067299	0.223438049	0.331996
COAD	FDFT1	CD8_naive	−0.11569	0.035950323	0.068571
COAD	FDFT1	Central_memory	0.122809	0.02591327	0.08246
COAD	FDFT1	Cytotoxic	0.031068	0.574448762	0.658329
COAD	FDFT1	DC	−0.03072	0.578734951	0.694377
COAD	FDFT1	Effector_memory	0.135348	0.014011419	0.040757
COAD	FDFT1	Exhausted	0.079108	0.15223709	0.238354
COAD	FDFT1	Gamma_delta	−0.09062	0.100848862	0.185006
COAD	FDFT1	InfiltrationScore	−0.12446	0.02396175	0.039701
COAD	FDFT1	MAIT	0.065499	0.236098889	0.305429
COAD	FDFT1	Macrophage	−0.09246	0.094058982	0.163471
COAD	FDFT1	Monocyte	−0.1559	0.004590939	0.01021
COAD	FDFT1	NK	0.039389	0.476457385	0.547267
COAD	FDFT1	NKT	−0.04362	0.430394011	0.526828
COAD	FDFT1	Neutrophil	−0.08117	0.141813904	0.21543
COAD	FDFT1	Tfh	0.046057	0.405031918	0.489801
COAD	FDFT1	Th1	0.151289	0.005967945	0.021079
COAD	FDFT1	Th17	0.105758	0.055320302	0.106168
COAD	FDFT1	Th2	−0.03301	0.550702397	0.616684
COAD	FDFT1	Tr1	−0.10243	0.063502854	0.11671
COAD	FDFT1	iTreg	−0.0131	0.81287067	0.867349
COAD	FDFT1	nTreg	0.201106	0.000241193	0.000564
COAD	HSPB1	Bcell	0.016112	0.770935023	0.823707
COAD	HSPB1	CD4_T	0.11569	0.035950057	0.055578
COAD	HSPB1	CD4_naive	0.033855	0.540600482	0.887885
COAD	HSPB1	CD8_T	0.009588	0.862452846	0.90608
COAD	HSPB1	CD8_naive	0.089716	0.104293081	0.16892
COAD	HSPB1	Central_memory	−0.10655	0.053513211	0.142356
COAD	HSPB1	Cytotoxic	0.050055	0.365454017	0.457639
COAD	HSPB1	DC	0.038725	0.48393356	0.611192
COAD	HSPB1	Effector_memory	3.41E-05	0.99950775	0.999508
COAD	HSPB1	Exhausted	−0.09535	0.084202724	0.146841
COAD	HSPB1	Gamma_delta	−0.00273	0.960653853	0.975942
COAD	HSPB1	InfiltrationScore	0.107573	0.051244212	0.078929
COAD	HSPB1	MAIT	0.115156	0.036820715	0.059061
COAD	HSPB1	Macrophage	0.066933	0.225973178	0.330497
COAD	HSPB1	Monocyte	−0.05647	0.307179661	0.39625
COAD	HSPB1	NK	0.102513	0.063276701	0.091338
COAD	HSPB1	NKT	0.117307	0.033419966	0.060221
COAD	HSPB1	Neutrophil	−0.07423	0.179239336	0.26232
COAD	HSPB1	Tfh	−0.01071	0.846477576	0.88167
COAD	HSPB1	Th1	−0.19059	0.000509289	0.002927
COAD	HSPB1	Th17	0.036326	0.511433304	0.622967
COAD	HSPB1	Th2	0.167214	0.002343114	0.004378
COAD	HSPB1	Tr1	−0.02527	0.647847324	0.737746
COAD	HSPB1	iTreg	−0.11985	0.02974044	0.062032
COAD	HSPB1	nTreg	−0.17044	0.001918694	0.003871
COAD	SLC1A5	Bcell	−0.24097	9.89224E-06	4.42E-05
COAD	SLC1A5	CD4_T	−0.31704	4.07515E-09	1.65E-08
COAD	SLC1A5	CD4_naive	0.055306	0.317257076	0.806027
COAD	SLC1A5	CD8_T	−0.20955	0.000128657	0.000662
COAD	SLC1A5	CD8_naive	0.13924	0.011461408	0.025583
COAD	SLC1A5	Central_memory	−0.11411	0.038573732	0.110974
COAD	SLC1A5	Cytotoxic	−0.15461	0.0049438	0.01088
COAD	SLC1A5	DC	0.022538	0.683790646	0.778397
COAD	SLC1A5	Effector_memory	−0.05282	0.339523746	0.48725
COAD	SLC1A5	Exhausted	0.160205	0.003572487	0.009971
COAD	SLC1A5	Gamma_delta	0.142781	0.009507343	0.026696
COAD	SLC1A5	InfiltrationScore	−0.25754	2.20581E-06	7.13E-06
COAD	SLC1A5	MAIT	−0.30951	9.81186E-09	5.85E-08
COAD	SLC1A5	Macrophage	−0.11209	0.04217849	0.083998
COAD	SLC1A5	Monocyte	0.267512	8.48819E-07	4.74E-06
COAD	SLC1A5	NK	−0.33351	5.47551E-10	2.67E-09
COAD	SLC1A5	NKT	−0.21394	0.000091869	0.000321
COAD	SLC1A5	Neutrophil	0.258419	2.03021E-06	9.46E-06
COAD	SLC1A5	Tfh	−0.33927	2.63682E-10	2.36E-09
COAD	SLC1A5	Th1	0.042495	0.442362903	0.583992
COAD	SLC1A5	Th17	−0.22282	0.000045489	0.00024
COAD	SLC1A5	Th2	−0.33281	5.97566E-10	2.8E-09
COAD	SLC1A5	Tr1	−0.15019	0.006347942	0.015927
COAD	SLC1A5	iTreg	0.094444	0.087197264	0.152343
COAD	SLC1A5	nTreg	0.326751	1.26625E-09	6.72E-09

Note: cor, correlation.

### 3.5 Prediction of FRGs’ regulation network

**FIGURE 4:**
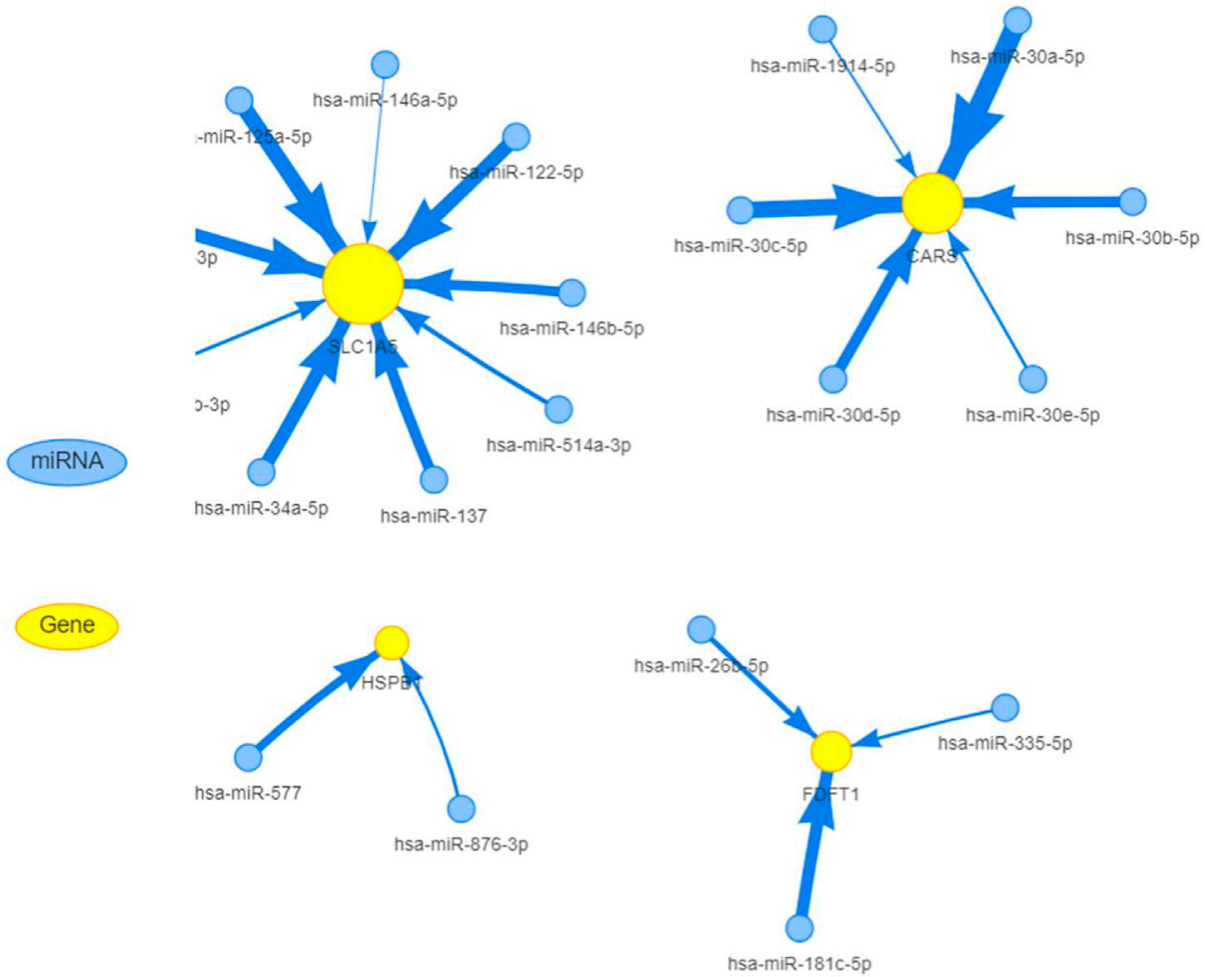
MiRNAs target FGRs in colon cancer. FGRs can be controlled by miRNAs in COAD.

MiRNAs have been linked to the regulation of tumor-associated genes in a variety of cancer types, although their role in the deregulation of FGRs remains uncertain. We initially identified the FGRs miRNAs network in COAD using the GSCALite tool. Figure 4 depicts evidence that miRNAs target FGRs identified by the GSCALite program. Using ChIP-seq datasets, we also discovered transcription factors that may posttranscriptionally modulate FGRs expression (Table 3). Hsa miR- 37–3p has experimentally verified to target ironoptosis-related SLC1A5 in melanoma (doi: 10.1038/s41418-017-0053-8). These consistent results suggest that anticipated miRNAs may have a role in COAD as well.

**TABLE 3:** Regulation of FGRs expression by transcription factors identified using Chip-Seq data.

Promoter	Promoter	Promoter	TF	Motif_ID	Motif	Motif	Motif	Score	P.Value
_Chr	_Start	_End			_Start	_End	_Strand		
chr11	3076681	3080681	TFAP4	Transfac.V$AP4_01	3077993	3078010	+	18.3577	5.74E-07
chr11	3076681	3080681	SP1	Transfac.V$SP1_Q6	3078348	3078360	−	16.4079	9.18E-07
chr11	3076681	3080681	SP1	Transfac.V$SP1_Q6_01	3078349	3078358	−	15.9857	6.78E-07
chr11	3076681	3080681	TFAP2C	Transfac.V$TFAP2C_01	3076752	3076763	+	15.7424	0.00000035
chr11	3076681	3080681	TFAP2C	Transfac.V$TFAP2C_01	3076752	3076763	−	16.7576	3.11E-08
chr11	3076681	3080681	TFAP2C	JASPAR2014.MA0524.1	3076752	3076766	−	17.5306	0.00000024
chr11	3076681	3080681	TFAP2C	Jolma2013.TFAP2C_DBD	3076752	3076763	+	16.6972	2.08E-07
chr11	3076681	3080681	TFAP2C	Jolma2013.TFAP2C_DBD	3076752	3076763	−	17.3853	3.11E-08
chr11	3076681	3080681	TFAP2C	Jolma2013.TFAP2C_full	3076752	3076763	+	15.6869	3.19E-07
chr11	3076681	3080681	TFAP2C	Jolma2013.TFAP2C_full	3076752	3076763	−	16.6869	3.11E-08
chr8	11658189	11662189	MYC	JASPAR2014.MA0147.2	11661289	11661298	+	15.5455	9.09E-07
chr19	47289842	47293842	NFYA	Transfac.V$NFYA_Q5	47291924	47291937	−	18.3026	6.79E-08
chr19	47289842	47293842	TFAP2C	Transfac.V$TFAP2C_03	47292525	47292535	+	15.1774	8.11E-07
chr19	47289842	47293842	TFAP2C	Jolma2013.TFAP2C_DBD_2	47292525	47292535	+	16.2755	6.13E-07
chr19	47289842	47293842	TFAP2C	Jolma2013.TFAP2C_full_3	47292525	47292535	+	15.1327	8.11E-07
chr7	75929874	75933874	TFAP4	Transfac.V$AP4_Q6_02	75932064	75932076	−	15.102	7.72E-07
chr7	75929874	75933874	TFAP4	Transfac.V$AP4_Q6_02	75932169	75932181	−	15.8265	2.75E-07
chr7	75929874	75933874	FOXP1	Transfac.V$FOXP1_01	75930287	75930306	+	17.5952	3.63E-08
chr7	75929874	75933874	FOXP1	Transfac.V$FOXP1_01	75931150	75931169	+	7.60714	0.00000053
chr7	75929874	75933874	HSF1	Transfac.V$HSF1_Q6	75932933	75932949	−	20.5688	7.59E-08
chr7	75929874	75933874	HSF1	Transfac.V$HSF1_Q6	75932940	75932956	+	17.9817	7.28E-07
chr7	75929874	75933874	SP1	Transfac.V$SP1_02	75932674	75932684	−	19	7.65E-08
chr7	75929874	75933874	SP1	Transfac.V$SP1_03	75933079	75933088	+	17.0674	7.52E-07
chr7	75929874	75933874	SP1	Transfac.V$SP1_05	75931838	75931848	+	16.5385	4.69E-07
chr7	75929874	75933874	SP1	Transfac.V$SP1_Q2_01	75932676	75932685	+	16.0921	6.78E-07
chr7	75929874	75933874	SP1	Transfac.V$SP1_Q4_01	75931837	75931849	−	16.9737	2.79E-07
chr7	75929874	75933874	SP1	Transfac.V$SP1_Q6	75931837	75931849	−	17.0132	0.0000005
chr7	75929874	75933874	SP1	Transfac.V$SP1_Q6_01	75932675	75932684	−	15.9857	6.78E-07
chr7	75929874	75933874	SP1	Transfac.V$SP1_Q6_02	75932195	75932211	+	15.2816	5.48E-07
chr7	75929874	75933874	SP1	Transfac.V$SP1_Q6_02	75932196	75932212	+	14.9029	8.55E-07
chr7	75929874	75933874	SP1	Transfac.V$SP1_Q6_02	75932198	75932214	+	16.3592	1.41E-07
chr7	75929874	75933874	SP1	Transfac.V$SP1_Q6_02	75932668	75932684	+	16.4806	0.00000012
chr7	75929874	75933874	SP1	Transfac.V$SP1_Q6_02	75932669	75932685	+	18.3883	7.35E-09
chr7	75929874	75933874	SP1	Transfac.V$SP1_Q6_02	75932671	75932687	+	14.9563	8.04E-07
chr7	75929874	75933874	SP1	Transfac.V$SP1_Q6_02	75932672	75932688	+	14.8786	0.00000088
chr7	75929874	75933874	SP1	Transfac.V$SP1_Q6_02	75932673	75932689	+	15.9466	2.41E-07
chr7	75929874	75933874	SP1	Transfac.V$SP1_Q6_02	75932674	75932690	+	17.8058	1.81E-08
chr7	75929874	75933874	HSF1	JASPAR2014.MA0486.1	75932935	75932949	−	18.9286	2.34E-07
chr7	75929874	75933874	HSF1	JASPAR2014.MA0486.1	75932940	75932954	+	18.7429	2.78E-07
chr7	75929874	75933874	TP63	JASPAR2014.MA0525.1	75931308	75931327	+	17.0577	0.00000077
chr7	75929874	75933874	SP1	Jolma2013.SP1_DBD	75931838	75931848	+	16.5	4.69E-07

Note: chr11 indicates CARS1 location, chr8 indicates FDFT1 location, chr19 represents SLC1A5 location, chr 7 represents HSPB1 location.

## 4 Discussion

Colon cancer is the most prevalent primary malignant tumor that is characterized by rapid growth and treatment resistance, resulting in significant mortality and morbidity. Iron death is a pattern of cell death caused by the accumulation of active oxygen species (ROS) of iron and lipids within cells and plays a vital role in tumorigenesis and development. (Dixon, 2017; Jiang et al., 2021). A number of studies have shown that ferroptosis can be used to treat cells that have developed resistance to drugs, which emphasized the importance of ferroptosis in the treatment of CRC patients (Friedmann Angeli et al., 2019; Wang et al., 2021). However, the underlying mechanisms and biomarkers of iron death in cancer remain to be studied. In the current study, we used bioinformatics and statistical methods to screen TCGA for information relevant to ferroptosis in COAD metastasis. We also created a risk for ferroptosis, which we believe could serve as a possible diagnostic biomarker. Using the TCGA data collection, we initially evaluated DEGs ferroptosis genes in primary and next in metastasis COAD patients. According to the findings of the Kaplan Meier survival analysis four genes having a significant correlation with OS. The prognostic accuracy of these ferroptosis-related genes in COAD patients was examined using univariate and multivariate Cox regression. Patients with COAD who were classified as high risk had a lower overall survival time than those who were classified as low risk. Studies have shown that fatal lipid peroxidation [20] is a cause of ferroptotic cell death (Gao et al., 2015). The accumulation of intracellular iron resulting from the depletion of ferritin or iron transporters and the subsequent peroxidation causes the formation of lipid peroxides and iron hypertrophy (Stockwell and Jiang, 2019). Four ferroptosis-related genes (FDFT1, HSPB1, SLC1A5, and CARS1 were identified in this investigation. Two genes involved in ferroptosis and lipid metabolism, FDFT1 and HSPB1, have been related to a poor prognosis in colorectal cancer, which is congruent with our own findings in COAD metastases (Liu et al., 2021; Yang et al., 2021). In addition to being increased in CRC, HSPB1 was also revealed to be related to worse survival in the present study (Wang et al., 2012; Konda et al., 2017). However, neither CARS1 nor SLC1A5 have previously been documented in CRC, therefore our results suggest that this area requires additional investigation. One of the most important regulators of cell survival is the PI3K/Akt/mTOR pathway. ROS can activate this pathway either by oxidizing kinases directly or by oxidizing the negative phosphatase regulators of this system, including phosphatase and tensin homolog (PTEN), protein-tyrosine phosphatase 1B (PTP1B), and protein phosphatase 2 (PP2) (PP2A). The effects of above mentioned FRGs on ROS can cause an increase in activity of the PI3K/Akt/mTOR signaling cascade. Furthermore, Curcumin’s anti-cancer effects in colon cancer cells are modulated by HSPB1’s ability to induce reactive oxygen species (ROS) generation and autophagy (Liang et al., 2018). The above results appeared to support the existing results that FRGs contributed to colon cancer progression and invasion *via* oxidative phosphorylation. FGRs may act as ROS-inducing agents and can activate TGFB, which is known to promote tumorigenesis, angiogenesis, and metastasis. Additionally, TGFB regulates the genes responsible for inflammation, cell proliferation, differentiation, and survival. Hypoxia is known to stimulate the production of mitochondrial reactive oxygen species (mROS), which in turn increases and stabilizes hypoxia-inducible factor-1 (HIF1a), which contributes to the survival and progression of tumors by upregulating the genes that regulate tumor angiogenesis and metastasis. On the other hand, several distinct DNA repair systems collaborate to decrease the risk that this damage may lead to dangerous mutations. There is a link between FRGs and genes involved in DNA repair pathways, allowing cells to be more resistant to DNA damage. FGRs showed positive correlation with a checkpoint control pathway and enhanced the activity of genes involved in DNA replication pathway that would normally prohibit cells from initiating DNA replication when breaks are present. Previous research reported that increasing SLC7A11 and GPX4 expression in HCT116 inhibited iron-induced lipid peroxidation and protected cells from ferroptosis (Yuan et al., 2021).

The infiltration of different types of immune cells is a principal determining factor for the immune response at primary and secondary tumor sites in the tumor microenvironment. We further analyzed the influence of CARS1 level on immune cell infiltration. The levels of FRGs significantly affects the infiltration level of B cells, CD4-T, CD8-T, central memory, NKT, Tfh, Th17, Th2, and Tr1cells. These results indicated that FRGs had a key regulatory effect on the immune cells in COAD patients. This is a preliminary exploration, but we intend to follow it up with experimental confirmation and biogenesis research.

Study limitation: All the findings in this manuscript were of speculation based on the transcriptional analyses using bioinformatics analysis. *In vitro* and *in vivo* investigations should be conducted to confirm the functional involvement in COAD. In conclusion, based on bioinformatics and statistical analysis, this study revealed associations between ferroptosis-related genes (FRGs) and colon adenocarcinoma (COAD). Prognostic models for ferroptosis-related programmed cell death in COAD metastasis were developed by identifying 4 signature genes, constructing a nomogram based on univariate and multivariate Cox regression model, and analyzing related pathways, immune cell infiltration, and regulatory networks.

## Data Availability

The original contributions presented in the study are included in the article/Supplementary Material, further inquiries can be directed to the corresponding authors.
